# Renal Disease in Cryoglobulinemia

**DOI:** 10.1159/000516103

**Published:** 2021-05-19

**Authors:** Thomas Menter, Helmut Hopfer

**Affiliations:** Pathology, Institute for Medical Genetics and Pathology, University Hospital Basel, University of Basel, Basel, Switzerland

**Keywords:** Glomerulonephritis, Structured deposits, Electron microscopy, Hepatitis C

## Abstract

**Background:**

Renal disease in cryoglobulinemia is difficult to grasp and diagnose because it is rare, serological testing is challenging and prone to artifacts, and its morphology is shared by other renal diseases resulting in a spectrum of differential diagnoses. On occasion, a definitive diagnosis cannot even be rendered after immunofluorescence and electron microscopic studies.

**Summary:**

Based on kidney biopsies seen in our routine diagnostic and referral practice, we discuss and illustrate various morphological patterns of renal injury associated with cryoglobulins. We outline key pathophysiologic and clinical aspects associated with cryoglobulinemia induced renal disease and describe morphologic changes with a focus on electron microscopy. We present our practical, morphology-based approach to diagnostic decision-making with special consideration of differential diagnoses and disease mimickers. Since cryoglobulins are rarely tested for prior to kidney biopsy, pathologists and clinicians alike must have a high level of suspicion when interpreting renal biopsies and managing patients.

**Key Messages:**

Cryoglobulinemia-associated glomerulonephritis (GN) is a multifactorial disease which is important to recognize for clinical practice. Morphological features suggestive of cryoglobulinemia-associated GN include a pattern of membranoproliferative GN with abundance of monocytes and the presence of (pseudo)thrombi. By electron microscopy, the main diagnostic features are a prominent infiltration of monocytes/macrophages and the presence of mesangial and subendothelial deposits with frequently curved microtubular/cylindrical and annular substructures.

## Disease Definition

Cryoglobulinemic glomerulonephritis (GN)/vasculitis refers to glomerular and/or vascular pathology in the clinical context of circulating cryoglobulins. Of note, it is not a morphological pattern or a specific immunohistochemical or electron microscopic finding that defines cryoglobulins, but a positive laboratory test, that is, immunoglobulins (Ig) that reversibly precipitate at temperatures <37°C. Laboratory testing is challenging, especially the temperature-sensitive pre-analytical phase is prone to mistakes, producing falsely negative results [1].

Depending on the Ig composition, Brouet et al. have classified cryoglobulins into 3 groups [2]. Type I cryoglobulins, 10–20% of the cases, are monoclonal Igs, commonly IgG, or IgM [3]. They occur in the context of monoclonal gammopathies, frequently in patients with overt lymphomas (esp. lymphoplasmacytic lymphoma/Waldenström's macroglobulinemia, multiple myeloma) or leukemia (most frequently chronic lymphocytic leukemia). Type II cryoglobulins are most frequently detected (50–65% of the cases) [3]. They consist of a monoclonal IgM and polyclonal IgG component (mixed cryoglobulins type II). Most often, the monoclonal IgM is directed against the Fc portion of the polyclonal IgG and is referred to as rheumatoid factor. Type II cryoglobulins are typically found in patients with hepatitis C infections but may also be detected in the setting of other infections or autoimmune diseases. Currently, hepatitis C accounts for 80–90% of these cases [4]. Type III cryoglobulins are composed of polyclonal IgM and IgG components (mixed cryoglobulins type III), make up 25–40% of cases, and are often found in autoimmune disorders or with various infections including hepatitis C [3]. Disease/GN caused by type III cryoglobulins is relatively uncommon. If no underlying condition/clinical association is found, cases are classified as so-called essential cryoglobulinemia.

While cryoglobulinemia indicates the detection of cryoglobulins in the serum, the term cryoglobulinemic vasculitis is used once clinical symptoms are present [4]. Pathologists frequently struggle with the diagnosis because cryoglobulins are rarely tested for prior to a kidney biopsy, follow-up information is often not provided in daily practice or test results are reported to be “negative.” Thus, pathologists may render cautiously worded diagnoses, such as “detection of an immune-complex mediated GN with a membranoproliferative pattern and deposition of structured deposits (see comment)” instead of diagnosing a “cryoglobulinemic GN.”

## Pathophysiology/Recent Findings

The pathogenesis of cryoglobulin-induced injury and disease is only partially understood. Cryoglobulinemic GN is characterized by intraglomerular deposition of immune complexes, complement activation, influx of leukocytes, and glomerular remodeling.

Aberrant B-cell function and lymphoproliferation are factors associated with the production of cryoglobulins. This has best been studied in patients with a hepatitis C virus (HCV) infection. HCV uses CD81 as cell-entry-receptor that is found not only on hepatocytes but also B-cells, and consequently, both cell types are infected. Infection of B-cells results in aberrant function and proliferation with release of mixed cryoglobulins into the circulation that also contains viral antigens [4]. While the precipitation of cryoglobulins at low temperature may explain the onset of vasculitis in the skin, it is less likely that low temperature plays an important role in the initiation of cryoglobulinemic GN. Rather, there is some experimental evidence that the amount of certain Ig subclasses may have an important role as shown in a mouse model of cryoglobulinemia [5]. In this model, IgG3-dominant immune complexes initiate GN independently of complement and Fc-receptors. Due to the longer and more flexible hinge region of mouse IgG3, much larger immune complexes can be generated at increased concentrations compared to IgG1-dominant or mixed IgG1-IgG3 complexes. Thus, it is conceivable that a sudden increase of the IgG3 concentration in glomerular capillaries due to ultrafiltration may contribute to proteinaceous thrombus formation seen in acute phases of cryoglobulinemic GN. This may be also true for human disease as the hinge regions of human IgG subclasses also differ in their hinge regions [6].

Complement consumption, in particular low serum levels of C4, is a key laboratory feature of cryoglobulinemic GN/vasculitis. Deposition of complement factor C3 in tissues is a typical finding by immunofluorescence microscopy. However, the role complement factors play in the pathogenesis of cryoglobulinemic GN/vasculitis is not fully understood since animal studies seem to suggest that cryoglobulin-induced renal injury appears to be less complement-dependent than other forms of GN [7].

Glomerular monocyte-/macrophage-infiltration (cells expressing CD68) is a prominent and characteristic finding in active cryoglobulinemic GN. While phagocytosis of the cryoglobulins poses no challenge to cells, intracellular/intralysosomal degradation of the immune complexes is impaired [8]. Glomerular influx of monocytes/macrophages also seems to confer a pro-inflammatory insult to the glomeruli [9]. An interesting open question is why glomerular lesions rarely show necrosis or crescent formation, whereas cryoglobulinemic leukocytoclastic vasculitis in the skin or the kidney more often presents with fibrinoid necrosis.

## Clinical Manifestations

Cryoglobulinemic GN often is an unexpected diagnosis. The most common clinical manifestations are (moderate) proteinuria, microscopic hematuria, and mild renal insufficiency that are all signs seen in many different glomerular diseases [10, 11, 12]. Monoclonal (type I) and mixed cryoglobulins (type II–III) differ somewhat in their clinical presentation. While type I cryoglobulinemia frequently causes symptoms related to vascular stenosis/occlusion at low temperature (e.g., Raynaud phenomenon, digital ischemia, livedo reticularis, and skin necrosis), type II and III cryoglobulinemia can present with waxing and waning nonspecific symptoms. Many patients present with the classical “Meltzer's triad” of skin purpura (most often on the legs), arthralgia, and weakness. Clinical signs that can raise the possibility of an underlying cryoglobulinemic GN include skin purpura, HCV infection, known autoimmune diseases, monoclonal gammopathy, or a hematological malignancy. If complement consumption is additionally noted, in particular low serum C4 levels, the chances of an underlying cryoglobulinemic GN are even higher.

Kidney involvement is seen in ∼29% of patients with cryoglobulinemia. While it is very frequent in patients with type II cryoglobulins (∼84%), it is rare with type I and III cryoglobulins (type I: ∼4%; type III: ∼11%) [13].

The disease course is often indolent. The treatment and prognosis of cryoglobulinemic GN depend on the severity and the underlying disease, that is, the lymphoma, HCV infection or autoimmune disease is commonly preferentially treated [4]. Immunosuppression is used in patients with a rapidly progressive course regardless of the etiology. Additional independent poor prognostic factors seem to be older age, severe concurrent infections, the number of vasculitis flares, pulmonary or gastrointestinal involvement, and the degree of renal failure [10, 12, 14, 15].

## Light Microscopy

**Fig. 1:**
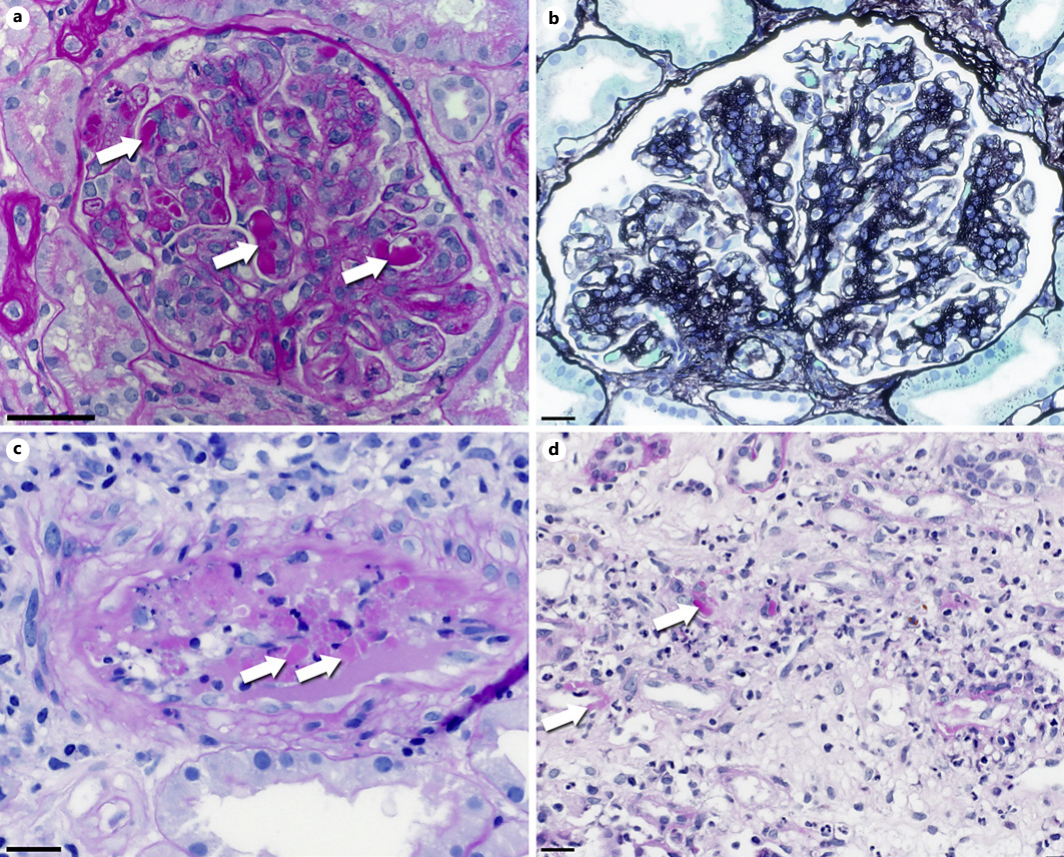
Light microscopy of cryoglobulinemic GN. **a** Early stage of MPGN in cryoglobulinemic GN with lobular architecture, prominent endocapillary thrombi (arrows), and hypercellularity. PAS stain. Original magnification 400-fold, bar = 50 µm. **b** Fully developed MPGN with expansion of the hypercellular mesangial matrix and double contours of the peripheral capillaries with cell interposition. The endocapillary cell proliferation is less pronounced. No thrombi. Methanamine stain. Original magnification 400-fold, bar = 20 µm. **c** Cryoglobulinemic vasculitis in an interlobular artery. Immune complexes are visible as PAS-positive material (arrows) in contrast to the PAS-negative fibrin precipitates. PAS stain. Original magnification 400-fold, bar = 20 µm. **d** Cryoglobulinemic capillaritis in the kidney medulla. A mixed, partly leukocytoclastic inflammatory infiltrate is visible within the interstitium. Some of the intact capillaries contain PAS-positive immune-complexes (arrows). PAS stain. Original magnification 400-fold, bar = 20 µm.

Mazzucco et al. [16] coined the term “cryoglobulinemic glomerulonephritis” in 1981 to emphasize some peculiarities of cryoglobulinemia-associated GN: striking endocapillary hypercellularity predominantly composed of monocytes, prominent duplication of peripheral basement membranes, and the presence of intracapillary (pseudo)thrombi. Thus, the most common morphologic pattern is that of a membranoproliferative GN (MPGN, Fig. 1a, b) [12, 17, 18, 19], while other patterns of glomerular injury, such as a mesangioproliferative GN or occasionally only minor abnormalities, also exist [17]. Different patterns do not reflect different disease entities, but rather mark disease severity, activity, and stage of the dynamic disease course.

Initially, proteinaceous hyaline thrombi consisting of immune complexes obstruct glomerular capillaries, usually in a focal and segmental distribution pattern (Fig. 1a). These thrombi are positive in the PAS stain and red in the Masson-Trichrome stain. Attraction of monocytes/macrophages and to a lesser extent polymorphonuclear leukocytes dominates the next stage accompanied by mesangial proliferation and duplication of capillary walls. The classical pattern of MPGN follows with a lobular architecture, global basement membrane doubling with cell interposition, and variable mesangial cell proliferation. Frequently, different patterns of glomerular injury can be seen side by side. Glomerular necrosis and crescent formation are uncommon features in cryoglobulinemic GN (<5% of cases) [20].

Vasculitis of interlobular arteries is present in up to one-third of the patients (Fig. 1c) [17, 21]. Infrequently, we have observed a capillaritis of the peritubular capillaries (Fig. 1d). In the interstitium, a lymphohistiocytic reactive inflammatory infiltrate can be found. Concomitant acute tubular damage is seen in a fifth to a fourth of cases. There is no tight correlation between histologic phenotypes and the type of circulating cryoglobulins [22].

## Immunofluorescence Findings/Immunohistochemistry

**Fig. 2:**
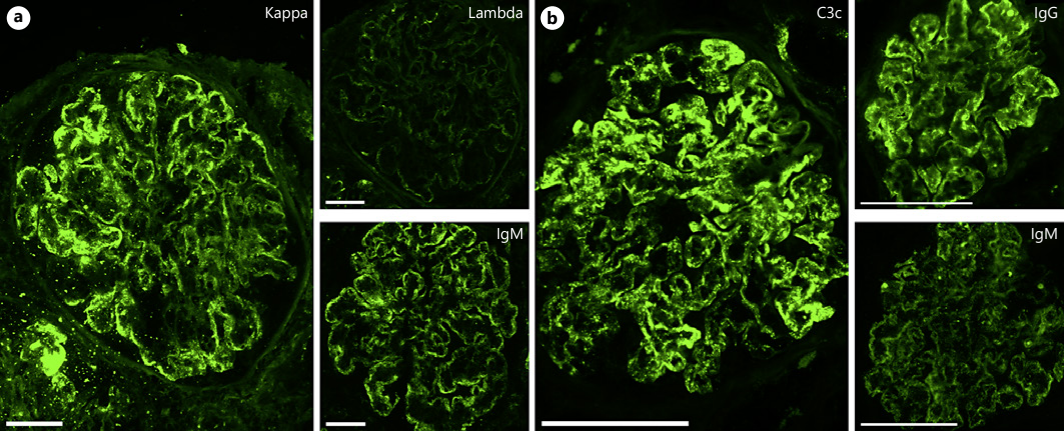
Immunofluorescence findings in cryoglobulinemic GN. **a** Type I cryoglobulins in a patient with splenic marginal zone ­lymphoma and viral hepatitis. The peripheral deposits consist of IgM-kappa. No lambda light chains are detected. Direct immunofluorescence. Original magnification 400-fold, bar = 50 µm. **b** Mixed cryoglobulins in a patient with Sjögren's syndrome. Mesangial and peripheral deposition of complement C3c, IgG, and to a lesser extent also IgM. Both kappa and lambda light chains were present (not shown). Direct immunofluorescence. Original magnification 400-fold, bar = 100 µm.

Immunofluorescence/immunohistochemical staining patterns vary according to the type of circulating cryoglobulins and the pattern of tissue injury. In biopsies with MPGN, both mesangial and peripheral granular deposits of Igs and complement factors are seen, whereas in cases with mesangial proliferations deposits are largely limited to mesangial regions. In cryoglobulinemia type I, monoclonal deposits of mainly IgG or IgM are noted staining for a monotypic light chain (Fig. 2a). This can be accompanied by staining for complement C1q and/or C3. In contrast, cases with mixed cryoglobulinemia typically show depositions of both IgG and IgM. Light chains are polyclonal and accompanied by complement factor C1q, C3, C4, and the membrane attack complex C5b-9. In these cases, expression of IgM and kappa light chains is usually dominant, reflecting the monoclonal nature of the IgM-component. IgG subclass deposition has not been investigated and reported in a systematic fashion. Only few studies have looked at IgG subclasses in serum cryoprecipitates. Type I cryoglobulins are most frequently restricted to IgG1 with few cases containing significant amounts of a second subclass. In contrast, cryoglobulins type II and III always contain several subclasses. Usually, IgG1 is the most prevalent subclass and the percentage of IgG4 is very low or absent [23].

## Electron Microscopy

**Fig. 3:**
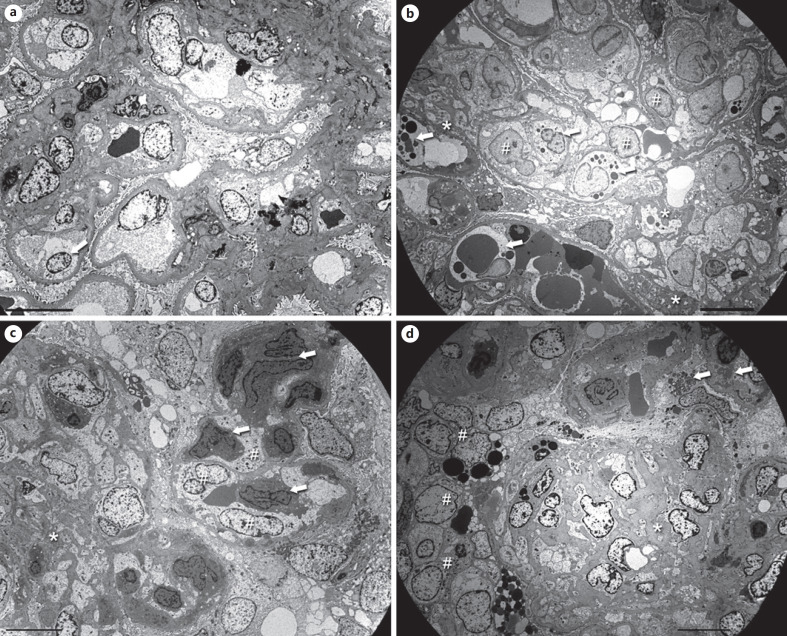
Electron microscopic findings in cryoglobulinemic GN at low power. **a** Mesangioproliferative cryoglobulinemic GN in a 61-year-old man with HCV-HIV-coinfection. The normal glomerular structures are preserved. The mesangial matrix is increased with an increase in the number of sectioned nuclei. The capillary lumina are open, and the endothelial cells are mildly swollen. In the lower left part, one endothelial cell nucleus is located in the periphery (arrow). Prominent monocyte/macrophage infiltration is not present. Original magnification 1,400-fold, bar = 10 µm. **b** Typical “nightmare” picture cryoglobulinemic MPGN in a 71-year-old woman with HCV-infection. Additional pictures of the same biopsy are shown in Figure 4d and f. Recognition of the lamina densa of the original basement membrane gives an overview. The image contains only little mesangial matrix (asterisks) infiltrated by macrophages. The capillary lumina are filled with swollen endothelial cells (hash signs) and many macrophages containing variable electron dense lysosomes (arrows). At this magnification, it is impossible to discriminate thrombi, deposits, and phagocytosed material. Even erythrocytes are difficult to differentiate. Original magnification 1,400-fold, bar = 10 µm. **c** A partly organized mesangiolysis (asterisk) on the left side of the picture in a 59-year-old man with HCV and a splenic marginal cell lymphoma. Within the mesangiolysis, it is impossible to differentiate the various cell types at this magnification. On the right side, a peripheral capillary is visible with massively swollen endothelial cells (hash signs) containing a more lucid cytoplasm compared to the monocytes in the lumen (arrows) as well as adjacent to the glomerular basement membrane. Original magnification 1,400-fold, bar = 10 µm. **d** A segmental sclerosis (asterisk) in the lower half of the picture in a 44-year-old woman with HCV-HIV-coinfection. In contrast to (**c**), the mesangiolysis shown here is older and contains much more mesangial matrix, and the macrophages have less cytoplasm. On the left side, a cellular crescent is visible (hash signs). The focus of the EM investigation should be the better-preserved capillaries seen in the upper right half. Some of the podocytes shown there contain myelin figures (arrows) raising the possibility of an additional Fabry's disease. Original magnification 1,400-fold, bar = 10 µm. HCV, hepatitis C virus.

**Fig. 4:**
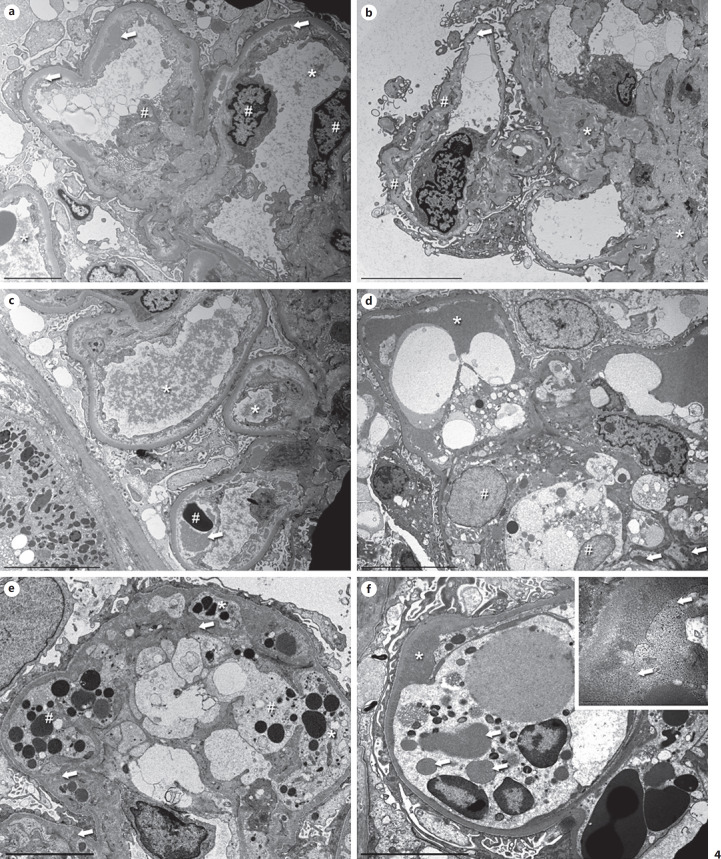
Characteristic EM features of cryoglobulinemic GN at medium power: MPGN pattern, endothelial cell swelling, monocyte/macrophage infiltration, remodeling of the GBM, and electron-dense deposits. **a** Biopsy of a 70-year-old woman with HCV and M. Waldenström. Pictures of the same biopsy can be seen in **c** and Figure 5e. Two capillary loops with typical MPGN features. The flocculent material in the lumina probably resembles precipitated cryoglobulins, especially the larger aggregates (asterisk). Other loops showed typical thrombi (see **c**). The endothelial cells (hash signs) are mildly swollen with a segmental loss of the fenestration. Beneath them, an irregular newly formed basement membrane is visible. The lamina rara interna is widened and contains subendothelial deposits of variable size (arrows). Only little cell interposition is visible, most likely extensions of the endothelial cells. Podocytes show a partial loss of their foot processes. Original magnification 3,500-fold, bar = 5 µm. **b** Biopsy of a 61-year-old woman with HCV. The mesangium is markedly expanded due to an increased matrix (asterisks). The peripheral capillaries show prominent remodeling with very thin stretches of GBM and splitting of the lamina densa, probably due to resolved deposits. Segmentally, cellular interposition is present in the periphery (hash signs). The lamina rara interna is mildly widened with few small residual subendothelial deposits (arrow). Original magnification 2,800-fold, bar = 10 µm. **c** Same biopsy as in (**a**) and Figure 5e. The flocculent material within the lumina forms vague thrombi (asterisks), which extends below the detached endothelium (arrow) in the capillary loop on the lower right side surrounding an erythrocyte (hash sign). There is a prominent activation of the endothelium. Mesangial and subendothelial deposits are present. Original magnification 2,800-fold, bar = 10 µm. **d** Biopsy of a 71-year-old woman with HCV. Pictures of the same biopsy are shown in Figure 3b and (**f**). A compact thrombus (asterisk) is seen within the capillary lumen with an intact endothelial cell layer. In the lower half, the cytoplasm of 2 macrophages contains multiple lysosomes of variable electron density (hash signs). The mesangium to the right contains several small mesangial deposits (arrows). Original magnification 2,200-fold, bar = 10 µm. **e** 4th sequential biopsy of a 79-year-old woman with cryoglobulinemia in the context of a thymoma. A picture of the same biopsy can be seen in Figure 5d. This extended peripheral capillary shows macrophages (hash signs) extending below the swollen endothelium (asterisk). The macrophages have a more lucent cytoplasm and contain multiple lysosomes, mostly filled with an electron dense material. Adjacent to the macrophages, few poorly formed deposits are present (arrows). The cytoplasm of the endothelial cell appears darker. A partly detached endothelial cell is visible in the middle of the lower edge. Original magnification 4,400-fold, bar = 5 µm. **f** Same biopsy as in Figure 3b and (**d**). This tangentially sectioned capillary is filled by a macrophage with multiple lysosomes of variable sizes. The material within some of the vesicles (arrows) has an identical appearance to the adjacent electron-dense deposits (asterisk). Original magnification 5,600-fold, bar = 5 µm. **Inset** A high magnification picture taken at a different location reveals a blurred interface between the extracellular deposits and the macrophage. In some areas, it is impossible to delineate the cell membrane (arrows). Original magnification 36,000-fold, bar = 500 nm. HCV, hepatitis C virus.

The key EM features are infiltration of monocytes/macrophages with prominent lysosomes, GBM duplication with cell-interposition, and electron-dense deposits in a mesangial and subendothelial location, which have a typical structured appearance in about half of the cases [18]. However, recognizing these features can be a challenge. While orientation is easy in cases with minor glomerular abnormalities or a mesangioproliferative pattern (Fig. 3a), the more common MPGN pattern is complex (Fig. 3b) with endothelial swelling, leukocyte infiltration, remodeling, deposits, occasionally proteinaceous thrombi, and/or signs of sclerosis (Fig. 3c, d). In these instances, the better-preserved capillaries should be the focus of the investigation. The best way to start is to look for the original lamina densa of the peripheral capillaries. This will help to outline the general structure of the glomerulus and give an idea of the localization of possible deposits. While in cryoglobulin-induced MPGN the outer aspect of the capillary wall is usually largely unchanged, the inner aspect undergoes remodeling with subendothelial new lamina densa formation/duplication, cell-interposition, and deposition of electron-dense deposits. Endothelial swelling is very common with a loss of fenestration, and in some cases positioning of endothelial cell nuclei to the periphery of the capillary loops (Fig. 3c, 4a, c, e, nuclei are usually located over mesangial zones). Widening of the lamina rara interna (Fig. 4a-c) and thinning of the lamina densa (Fig. 4b) can also be found. Some of these EM findings overlap with those seen in thrombotic microangiopathies.

Since proteinaceous thrombi usually have a focal and segmental distribution pattern, they may be underrepresented in electron microscopic studies due to sampling. If present they are quite variable in terms of shape, structure, and electron density (Fig. 4a, c, d) and resemble other deposits seen in the mesangium or the periphery (Fig. 4c). Higher magnification of deposits and microthrombi often shows a tubular and annular organoid substructure of many (but not all) deposits (see below).

Infiltration of monocytes/macrophages is common and frequently pronounced (Fig. 3b, c, 4d-f). These cells are located within the lumina, can migrate through the endothelial cell layer (Fig. 4e), and may also be found within the mesangium. Thus, the macrophages are responsible for most of the cellular interposition seen in cryoglobulinemic GN, compared to mesangial or endothelial cell interposition seen in other forms of MPGN. These macrophages contain extended lysosomes filled with electron dense material post-phagocytosis of intraluminal proteinaceous material (Fig. 3b-d, 4e, f). On occasion, even intralysosomal electron dense products may show a microtubular substructure.

**Fig. 5:**
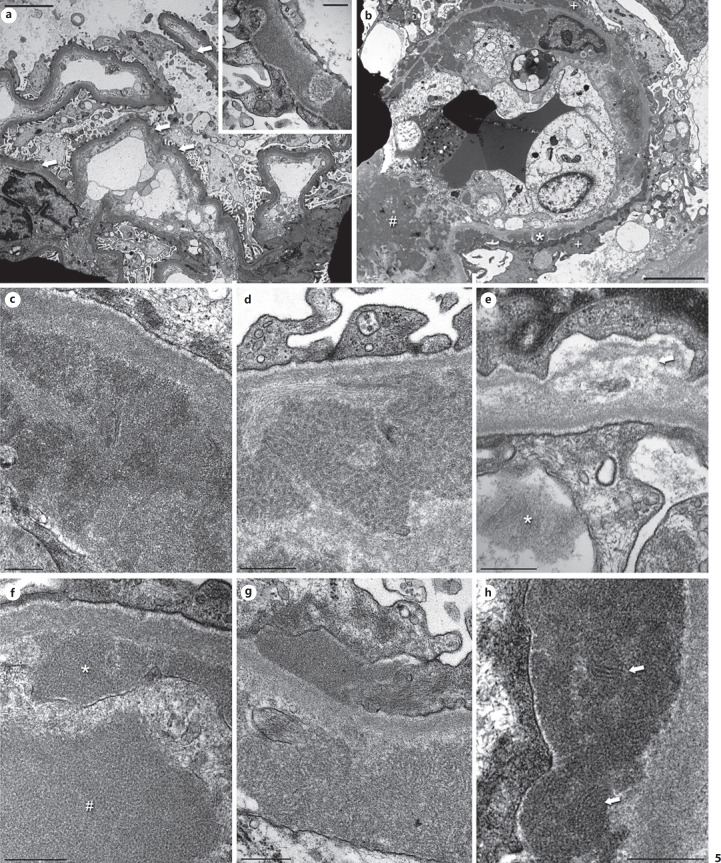
Localization and different quantity and quality of deposits in cryoglobulinemic GN. **a** Biopsy of an 85-year-old woman with HCV and presence of cryoglobulins type II. This medium magnification shows an overall normal architecture of the capillary loops, yet the peripheral basement membranes are mildly thickened and contain several partly dissolved intramembranous deposits (arrows). The endothelium is activated. Original magnification 2,800-fold, bar = 5 µm. **Inset** Higher magnification of some deposits without substructures. Original magnification 36,000-fold, bar = 500 nm. **b** Biopsy of a 19-year-old woman with a known history of SLE, cryoglobulinemia, and increasing disease activity. In contrast to (**a**), this capillary loop contains abundant subendothelial (asterisk), subepithelial (plus signs), and mesangial deposits (hash sign), some of which are dissolving. The peripheral basement membranes show duplication with matrix and cellular interposition (arrow) as well as massively swollen endothelial cells, which lead to a markedly narrowed lumen. The podocytes show extensive foot process effacement. Original magnification 3,500-fold, bar = 5 µm. **c** Biopsy of a 55-year-old woman with essential cryoglobulinemia. This amorphous deposit shows some structures which are reminiscent of tubules and curvilinear structures, yet they are not well enough formed to consider them as structured (compare to **d**). Original magnification 28,000-fold, bar = 500 nm. **d** 4th sequential biopsy of a 79-year-old woman with cryoglobulinemia in the context of a thymoma. A picture of the same biopsy can be seen in Figure 4e. Prototypical annular and tubular curvilinear substructures of a deposit. The diameter of the structures was 22 nm. This is a very characteristic feature of nonlupus cryoglobulinemic GN. However, these types of deposits are only present in about 50% of the patients. Original magnification 36,000-fold, bar = 500 nm. **e** Biopsy of a 70-year-old woman with HCV and M. Waldenström. Pictures of the same biopsy can be seen in Figure 4a and c. High magnification of a small thrombus with flocculent material in the capillary lumen consisting of fibrils (asterisk). The endothelial cells are activated, adjacent to the mesangial cell in the upper part, there is focal resorption and subsequent edema of the mesangial matrix (arrows). Original magnification 36,000-fold, bar = 500 nm. **f** Biopsy of a 74-year-old man with lymphoplasmacytic lymphoma. High magnification of a deposit with annular substructure (asterisk), partly within the cytoplasm of an adjacent macrophage (hash sign). Original magnification 36,000-fold, bar = 500 nm. **g** Biopsy of a 9-year-old girl with type III cryoglobulinemia in the context of an IgA-dominant postinfectious glomerulonephritis. High magnification of a partly amorphous, partly structured subepithelial deposit and a structured mesangial deposit consisting of fibrils with a diameter of 15–25 nm. Original magnification 28,000-fold, bar 500 nm. **h** Same biopsy as in (**b**). At high magnification, fingerprint-like tubular substructures (arrows) can be visualized in this subendothelial deposit. This is a typical finding in patients with lupus nephritis and has been described both with and without cryoglobulinemia. Original magnification 28,000-fold, bar = 500 nm. HCV, hepatitis C virus.

Although electron-dense deposits are a key feature of cryoglobulinemic GN, their amounts can vary considerably from case to case ranging from few in some patients with only minor abnormalities seen by light microscopy (Fig. 5a) to abundant in other biopsies (Fig. 5b). Most cases will have both subendothelial and mesangial deposits. Intramembranous and subepithelial deposits are less frequently present (<25% of cases) and often inconspicuous. In our experience, almost all biopsies show at least some amorphous deposits, that is, there is no structure to the deposits at high magnification (Fig. 5c). About half of the cases contain structured deposits (also termed “crystalloid” in the earlier literature; Fig. 5d-h), but the reported frequencies vary greatly. Depending on the direction the deposits are cut, they have a curved microtubular/cylindrical or annular structure (Fig. 5d, f). If measured, the tubules mostly have a diameter between 10 and 25 nm [20]. In patients diagnosed with cryoglobulinemic GN, identical structures can be observed in their cryoprecipitated serum, suggesting that the physicochemical properties of the involved Igs are responsible for the structured pattern [24]. Rarely, deposits may have a finely fibrillary substructure (Fig. 5e, g). In the context of lupus nephritis, cryoglobulin-associated deposits can resemble fingerprints (Fig. 5h). From a practical point of view, structured deposits are a characteristic finding in cryoglobulinemic GN, but in a routine diagnostic setting, they may be difficult to find, especially if deposits are sparse. If cryoglobulins are reported clinically, even amorphous deposits lacking a substructure but found in the setting of a suggestive overall morphologic phenotype might suffice for a clinicopathologic-correlation-diagnosis of “cryoglobulinemic GN.” If this is not the case, the overall pattern of tissue injury will be enough to recommend further testing for cryoglobulins.

**Fig. 6:**
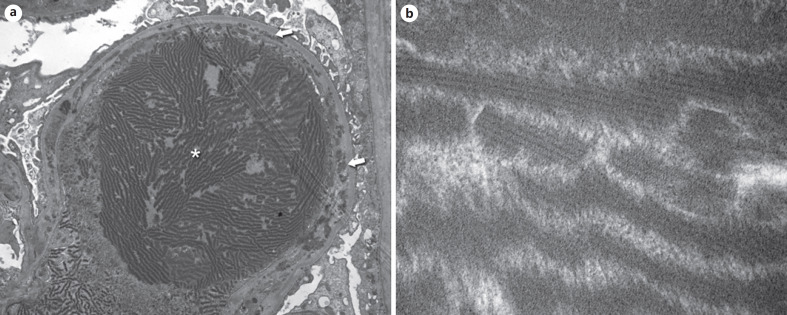
Electron microscopic findings in cryocrystalglobulinemia. **a** Biopsy of a 64-year-old woman with type I cryoglobulinemia in the context of kappa light chain myeloma. A glomerular capillary is occluded by a thrombus consisting of mostly structured electron dense material arranged in thick parallel bundles (asterisk). The endothelial cells are intact but have lost their fenestration. There is a duplication of the glomerular basement membrane with frequent subendothelial deposits (arrows) and cell interposition. Original magnification 7,200-fold. **b** High magnification reveals a crystalline substructure with a regular periodicity of more and less electron dense areas within the bundles. Original magnification 140,000-fold.

Rarely, type I cryoglobulins can assemble into crystalline arrays, which can be localized extra- or intracellularly (Fig. 6a, b). This is referred to as cryocrystalglobulinemia and typically presents with severe multi-organ involvement [25]. Intravascular crystal formation results in endothelial damage with subsequent thrombotic microangiopathy (TMA). Podocytes show variable findings ranging from well-preserved foot processes in few cases to extensive loss of foot processes in about one-third of the cases.

## Differential Diagnosis

Focusing on the EM features, the main differential diagnoses include other diseases with structured deposits and intracapillary protein thrombi (Table 1). In our reports, we regularly ask for cryoglobulin testing in cases with structured deposits or other prominent features of cryoglobulinemic GN. In addition, we recommend tests for a hepatitis C infection and in certain cases for underlying monoclonal gammopathies. Cryoglobulin testing should be considered in cases presenting with an otherwise unexplained MPGN pattern whereas cryoglobulinemic GN with minor changes such as mesangioproliferation are much more difficult to identify/diagnose because these lesions usually have other more common etiologies.

While the distribution of amyloid fibrils is quite different and does not cause great diagnostic challenges, fibrillary deposits seen in fibrillary GN might enter the differential diagnosis. However, on close inspection in those cases the deposits are exclusively constituted of fibrils with a random arrangement, location within the mesangial matrix, deep in capillary walls infiltrating the lamina densa, and immunohistochemical positivity for DNAJB9 [26]. Fibronectin glomerulopathy only vaguely resembles cryoglobulinemic GN and does not constitute a great diagnostic challenge [27].

Immunotactoid GN is defined by the presence of microtubular structures with a diameter of usually >30 nm (range 14–90 nm), thus, they are commonly larger than cryoglobulins [28, 29, 30]. Immunotactoid GN is associated with a paraprotein or hematologic malignancy in two-thirds of the cases [31]. The microtubules are longer with often a typical arrangement in parallel bundles. Additional amorphous deposits are minimal or absent, protein thrombi, infiltrating monocytes/macrophages are not observed in large numbers, and engulfed intracellular deposits are not detected. As in cryoglobulinemic GN, deposits are found in the mesangium, the subendothelial space and are not seen within the lamina densa. While cryoglobulins are rarely seen subepithelially, immunotactoid deposits frequently also have a subepithelial distribution pattern. Work by Herrera et al. comparing cases of immunotactoid and cryoglobulinemic GN suggested a significant overlap based on morphology, clinical presentation of the patients (presence of [mostly monoclonal] cryoglobulins in 3/7 patients with immunotactoid GN) and mass spectrometry [32]. In contrast, a recent series of 73 patients showed no signs of cryoglobulinemia or cryoglobulinemic vasculitis associated with immunotactoid GN [33].

While cryoglobulinemic GN has a characteristic morphological pattern, it is important to note that it is not a homogeneous disease category. Therefore, a careful clinicopathological correlation is necessary to identify underlying/associated diseases. In several of these (e.g., IgA-dominant infectious GN, MPGN with masked monoclonal Ig deposits, lupus nephritis; see below), only few or some of the patients have cryoglobulinemia while the cryoglobulin negative patients can still show identical morphological patterns in their kidney biopsies.

IgA-dominant infectious GN has been recognized as an important post-/peri-infectious pattern in kidney biopsies in the last 2 decades [34, 35, 36, 37]. It is frequently, but not always, associated with staphylococcal infections and often seen in diabetic patients. One of its defining features is codominant IgA staining by immunofluorescence or immunohistochemistry. Morphologically, various patterns have been described by light microscopy, intracapillary thrombi are present in some of the cases and rarely structured deposits have been recognized by EM. Cryoglobulin testing has revealed at least transient positivity in some of these cases suggesting that the immune complexes can have cryoglobulin activity contributing to glomerular injury. We recommend classifying such cases as IgA-dominant infectious GN and comment on the possibility/existence of a cryoglobulin component. In contrast, classical postinfectious GN due to streptococci is a less challenging differential diagnosis. It usually exhibits the well-known EM pattern with prominent large subepithelial humps, few small and scattered subendothelial deposits and, depending on the timing of the biopsy, mesangial deposits. Infectious GN due to endocarditis, infection of a cerebrospinal shunt, abscesses, or osteomyelitis are more difficult to recognize as the EM changes are much more variegated. In contrast to cryoglobulinemic GN, thrombi, structured deposits, and prominent macrophages engulfing the electron dense material are not seen in these cases.

MPGN with masked monotypic Ig deposits is a recently recognized paraprotein-associated entity characterized by monoclonal deposits, most frequently IgG kappa, which is not detected by routine immunofluorescence but is visible only after enzyme digestion of formalin-fixed and paraffin-embedded tissue [38]. By EM, some cases showed thrombi, deposits with substructure and phagocytosis of deposits by macrophages suggesting cryoglobulin activity, which was indeed documented in one of the patients.

Patients with systemic lupus erythematosus frequently also have cryoglobulinemia (often type III), but apparently, this has little impact on the course of lupus nephritis [39, 40]. Compared to the average cryoglobulinemic GN, kidney biopsies in lupus nephritis contain more electron-dense deposits and much less monocytes/macrophages. Tubuloreticular structures are another typical finding of lupus nephritis [41]. Whether the “fingerprint-substructures” seen in some deposits by EM hint at the deposition of cryoglobulins is debated. In support are 2 studies showing identical substructures by EM of both cryoprecipitated serum and glomerular electron-dense deposits in the corresponding biopsies [42, 43]. However, cryoglobulins could not be detected in other lupus patients with very similar biopsy findings [44]. This is not too surprising because the formation of structured deposits and cryoglobulinemic activity both depend on the physical and chemical properties of the immune complexes and the 2 features do not necessarily have to be linked to each other. From a practical point of view, these biopsies should be categorized as lupus nephritis.

Several EM features of cryoglobulinemic GN are overlapping with changes typically seen in cases of chronic TMA: endothelial swelling, widening of the lamina rara interna, and thrombi. However, a TMA does not show deposits and has, with the exception of fibrin, an unrevealing immunohistochemical/immunofluorescence staining pattern with a usual panel of antibodies.

Cryoprecipitates composed of fibrinogen, fibrin, and/or its degradation products are referred to as cryofibrinogen. Very few cases with kidney involvement have been reported. In contrast to cryoglobulinemic GN, no immune complexes are present. Instead, staining for fibrinogen/fibrin is detected. On TEM, the deposits show a unique structure consisting of large tubules with a central bore [45].

## Conclusion

Cryoglobulinemic GN is one of the most fascinating forms of GN because the glomerular lesions are highly variable depending on the phase of disease activity and remission including healing of glomerular injury. While the definition of cryoglobulins does not rely on morphology, the morphological findings are characteristic in cases presenting with an MPGN pattern, a prominent influx of monocytes/macrophages, and mesangial and subendothelial deposits that may show a microtubular substructure. Thus, morphology is an important hint for the diagnosis in many patients. Except for cryoglobulinemia type I, pathology does not help to identify the underlying etiology in most cases. Since cryoglobulins are rarely tested prior to kidney biopsy, pathologists must have a high level of suspicion and communicate the possibility of this rare type of GN in appropriate cases.

## Conflict of Interest Statement

The authors have no conflicts of interest to declare.

## Funding Sources

The authors did not receive any funding.

## Author Contributions

T.M. and H.H. each drafted sections of the manuscript, selected the individual figures, and wrote the figure legends. H.H. prepared the figure composites. Both edited and revised the manuscript and figures.
